# The financial crisis, health and health inequities in Europe: the need for regulations, redistribution and social protection

**DOI:** 10.1186/s12939-014-0058-6

**Published:** 2014-07-25

**Authors:** Roberto De Vogli

**Affiliations:** 1 School of Medicine, Department of Public Health Sciences, University of California Davis, One Shields Ave. Med Sci 1-C Build, Davis 95616, CA, USA

**Keywords:** Great recession, Health, Europe, Financial crisis, Inequality, Neoliberalism, Austerity and Global Health

## Abstract

In 2009, Europe was hit by one of the worst debt crises in history. Although the Eurozone crisis is often depicted as an effect of government mismanagement and corruption, it was a consequence of the 2008 U.S. banking crisis which was caused by more than three decades of neoliberal policies, financial deregulation and widening economic inequities.

Evidence indicates that the Eurozone crisis disproportionately affected vulnerable populations in society and caused sharp increases of suicides and deaths due to mental and behavioral disorders especially among those who lost their jobs, houses and economic activities because of the crisis. Although little research has, so far, studied the effects of the crisis on health inequities, evidence showed that the 2009 economic downturn increased the number of people living in poverty and widened income inequality especially in European countries severely hit by the debt crisis. Data, however, also suggest favorable health trends and a reduction of traffic deaths fatalities in the general population during the economic recession. Moreover, egalitarian policies protecting the most disadvantaged populations with strong social protections proved to be effective in decoupling the link between job losses and suicides.

Unfortunately, policy responses after the crisis in most European countries have mainly consisted in bank bailouts and austerity programs. These reforms have not only exacerbated the debt crisis and widened inequities in wealth but also failed to address the root causes of the crisis. In order to prevent a future financial downturn and promote a more equitable and sustainable society, European governments and international institutions need to adopt new regulations of banking and finance as well as policies of economic redistribution and investment in social protection. These policy changes, however, require the abandonment of the neoliberal ideology to craft a new global political economy where markets and gross domestic product (GDP) are no longer the main national policy goals, but just means to human and health improvements.

## Introduction

In 2009, Europe was hit by one of the worst sovereign debt crises in recent history. The crisis started in Greece when the new government revealed that previous administrations (helped by investment bank Goldman Sachs) had been misreporting budget data [[1]]. From Greece, the crisis spread to other European economies, especially Portugal, Ireland, Italy and Spain, the so-called PIIGS. In the following years, most European nations experienced rapid rises of unemployment, widening inequities in wealth and political instability. The aim of this article is to examine the consequences of the Eurozone crisis in terms of health and health inequities. Before examining the health effects of the economic downturn, however, it is important to first examine the policies that created the conditions for the crash and the underlying ideology upon which these policies are based.

## The causes of the crisis: neoliberal ideology, financialization and inequities in wealth

Although mainstream media outlets often portray the Eurozone crisis as an effect of government corruption, mismanagement of public finance and inefficient labor markets, in reality the downturn was largely an effect of the 2008 U.S. banking crisis which was caused by more than three decades of neoliberal policies, deregulation, financialization and widening economic inequities.

It is important to remember that the two decades after World War II were characterized by government interventions in market affairs and stringent regulations of banks and finance. In the US, the banking industry was still regulated by the Glass-Steagall Act, passed by Roosevelt in 1933 as a response to the Great Depression. The bill created a wall separating investment banks from commercial banks, and insurance companies, and required banks to hold specific levels of cash reserves [[2]]. Regulations were stronger not only in the US, but also internationally with the fixed exchange rates and capital controls of the Bretton Woods System. By the beginning of the 1980s, however, the “neoliberal revolution” began and the regulations that safeguarded economic stability in the US and worldwide were dismantled one after another.

In the US, Reagan led the “deregulation crusade” first by eliminating regulations on the savings and loans industry (1982 Garn-St. German Depository Institutions Act) and then by introducing “securitization” that allowed investment banks to buy mortgages, pool them, and resell them in slices with varying levels of risk (1984 Secondary Mortgage Market Enhancement Act). The Clinton administration equally contributed to escalating financial deregulation. In 1999, he repealed the 1933 Glass-Steagall Act (the Gramm-Leach-Bliley Act) and all bans against single-stock futures. One year later his administration prohibited federal regulations of over-the-counter derivatives including the infamous mortgage-backed securities (2000 Futures Modernization Act) [[3]].

The end of the Bretton Woods System created a playing field for the rapid, undisturbed and untaxed movement of speculative capital worldwide, and also indirectly encouraged the concomitant proliferation of offshore financing in “tax havens” such as the Cayman Islands, Andorra, Monaco, Bermuda, and Switzerland [[3]]. This resulted in a “race to the bottom” where countries have been under pressure to lower tax rates of transnational corporations and wealthy individuals for fear of capital flight. It has been estimated that there is between $21 and $32 trillion hidden in “tax havens” and that this amount of unreported wealth would have generated income tax revenues of between $190-280 billion a year.

The undoing of the New Deal’s and Bretton Woods System’s most important regulations caused a radical transformation of the world economy that became dominated by a proliferation of new financial products such as collateralized debt-obligations, credit-default swaps, and mortgage-backed securities. In 2010, two years after the financial collapse, off-exchange trading of financial derivatives that included the toxic assets that caused the U.S. and E.U. banking crises was estimated at $601 trillion. This is quite remarkable because the total value of all goods and services produced in the same year – or the global gross domestic product (GDP) – was $63 trillion [[4]].

The indiscriminate proliferation of these complex financial instruments that both created and burst the housing bubble did not concern most neoliberal ideologues, however. The proponents of “financial innovation” justified the introduction of these risky instruments on the ground that they were conducive to stability and wealth creation [[5],[6]]. Most adhered to the neoliberal doctrine of the “self-correcting market”, or the assumption that unfettered free markets always produce optimal outcomes when they are not distorted by government interventions. This ideology, however, proved to cause financial instability and inequitable distribution of wealth and income.

**Figure 1:**
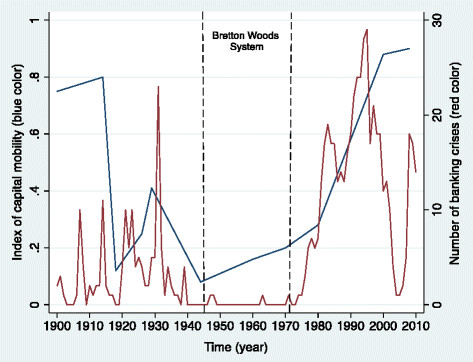
From the Great Depression to the Great Recession: Index of Capital Mobility and Number of Banking Crises in 69 Countries, 1900–2010.

Figure 1 shows temporal trends (1900–2010) in the frequency of banking crises and an index of international capital mobility in 69 countries [[7]]. As for the 1929 Great Depression, the 2008 Great Recession was anticipated by a rapid rise of global capital flow [[7]] and deregulation of financial markets [[8]]. It also shows that the frequency of banking crises was very low during the post-war period (1945–1971), a time influenced by the New Deal in the U.S. and capital controls and regulations of the Bretton Woods System worldwide. The progressive elimination of these regulations since the 1970s, triggered by the rise of neoliberal ideology, however, coincided with a rapid rise of banking crises in the 1980s and 1990s and created the circumstances for the 2008 global financial crash.

**Figure 2:**
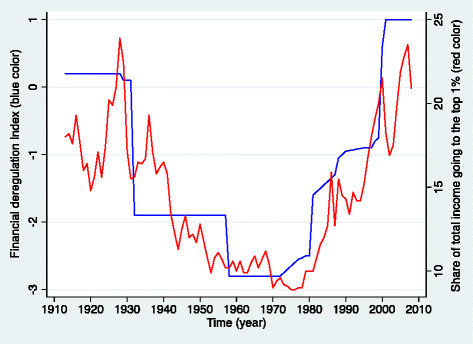
**The fall and rise of financial deregulation and share of total income Going to the top 1% in the United States, 1913–2008.**

The macroeconomic conditions that preceded the 1929 Great Depression and the 2008 Great Recession were not only characterized by indiscriminate international capital movement and deregulation, but also by a high concentration of wealth and income in the top distribution. The two crashes were preceded by decades of stagnating workers’ wages and spectacular profits for the so-called “top 1%” - rich investors, investment bankers and corporate executives - especially in the US where both originated. As shown in Figure 2, over the last century, the percentage of total income going to the top 1% of the U.S. population mirrored fluctuations over time of a “financial deregulation index” [[9]]. Peaks of economic inequities coincided with peaks of excessive deregulation of financial markets and the two greatest global crises in recent history.

Multiple authors analyzed the causal nexus between inequality and crises [[10],[11]] and even IMF economists have recently argued for restoring the bargaining power of low income groups as the most effective strategy to prevent future meltdowns [[12]]. The logic goes like this: as more money flows to the top social classes in a time of widening income gaps, the rich escalate their spending and induce the rest of society to follow suit. However, widening income inequalities are also associated with stagnation or decline in real value of workers’ wages. This forces low- and middle-income workers to over-borrow money to keep up with the consumerist lifestyle of the rich or to simply cover their material needs. It is no coincidence that the 2008 financial collapse was preceded by the rapid rise of consumer debt [[12]]. Times of widening inequalities also provide the wealthy with larger amounts of surplus capital to invest in short-term gains and highly speculative financial assets. Together with heavy borrowing at the bottom of the income distribution, over-investment in short-term profiteering of the super-rich promotes price distortions and asset bubbles, especially when governments no longer regulate financial markets. These price distortions and bubbles are obviously destined to burst since the prices of assets cannot increase forever. This can eventually lead to financial meltdowns as it happened after the 1929 stock crisis and the 2008 banking crisis in the U.S.

## The effects of the crisis: neoliberal austerity, suicides and inequities in health

**Figure 3:**
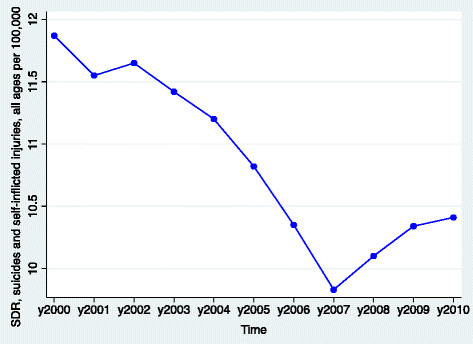
Standardized death rates in suicides and self-Inflicted injuries per 100,000 in 41 European Nations before and during the Great Recession, 2000–2010.

Although the crisis was mainly caused by wealthy investors, large banks, government institutions (e.g. credit rating agencies) and elites supporting policies of financial deregulation, [[13]] the effects were felt by low-income workers, small employers and the poor. Since the eruption of the global financial meltdown in 2008, about 10.2 million people in Europe became unemployed [[14]] and countless small businesses filed for bankruptcy. At the same time, the rise of public debts and the pressure of the European Commission, European Central Bank and the IMF, the so-called “Troika”, forced governments to borrow loans in exchange of austerity policies that further reduced access to basic human services especially among the poor. The combination of these economic shocks and reforms proved to be devastating for some. As shown in Figure 3, after almost a decade of steady decline, suicide rates in Europe shifted direction and rose quite rapidly for at least three years from 2008 to 2010 after the onset of the financial downturn and austerity policies.

In Greece, a country deeply affected by the crisis, suicides increased by 17% from 2007 to 2010 [[15]] with suicide attempts significantly elevated among persons with high scores of economic distress [[16]]. In the UK, between 2008 and 2010 there were an estimated 846 more suicides among men and 155 more among women than expected based on historical trends [[17]]. In Italy, between 2008 and 2010, there were about 290 suicides and attempted suicides in excess attributable to the crisis [[18]] and the fall of GDP per capita and the rise of unemployment resulted in a sharp increase of deaths due to mental and behavioral disorders especially among the elderly [[19]].

Little research has, so far, examined the effects of the financial crisis on inequities in health. However, some studies analyzed the impact of the economic downturn on two major determinants of health equity: poverty and income inequality. According to a report of the Organization for Economic Development and Cooperation (OECD), although average income of the top 10% income households in the OECD nations in 2010 was, more or less, similar to that of 2007, the income of the bottom 10% in 2010 was lower than that of 2007 by 2% per year. As a result, the number of people living in poverty in the OECD nations between 2007 and 2010 increased. The same report showed that “income inequality increased more in the first three years of the crisis…than it had in the previous 12 years” and that the rise in inequality was particularly apparent in countries that implemented deep budget cuts such as Ireland, Spain, Greece and Italy [[20]].

While financial crises are associated with rising unemployment, social instability and the rise of suicides, evidence also shows that they can paradoxically result in health improvements, on average. Although the 2008 economic downturn led to increased suicides in, there has also been an overall reduction of all-cause mortality rates. Clearly, the crisis produced differential health effects in different socioeconomic groups. It was deadly for some vulnerable sectors of society, but not necessarily harmful for other population sub-groups [[21]–[26]]. These favourable trends remain a puzzle, but it is widely accepted that during economic recessions road traffic fatalities decline. Other potentially favorable health effects from the crisis include increases of leisure time which, in turn, can lead to health- and wellbeing-enhancing activities among people who are relatively well off.

**Figure 4:**
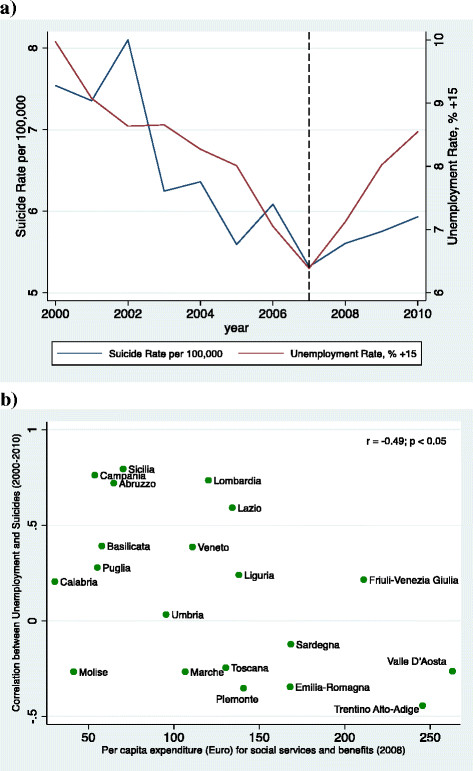
Temporal association between unemployment and suicides from 2000 to 2010 in Italy (a) and correlation between unemployment and suicides by Expenditure on Social Services and Benefits per capita in 20 Italian regions (b).

However, there are specific circumstances that facilitate positive health outcomes in times of crises: strong social protection and low economic inequities. A cross-national longitudinal analysis on the effects of the financial crisis showed that in countries with low social investment such as Spain, there was a positive correlation between unemployment and suicide, but this was not the case in countries with strong social protection such as Sweden, where a sharp increase of unemployment resulted in suicide reduction [[27]]. A similar picture emerged from a study examining the effect of unemployment on suicides in Italian regions with different levels of social investment (see Figure 4). Although rises of unemployment were strongly correlated with increases of suicides during the study period (from 2000 to 2010), the strength of the association between unemployment and suicide was negatively correlated with levels of investment in social welfare: in Italian regions investing more than 135€ per capita on social services such as Trentino-Alto Adige, a rise in unemployment was associated with a reduction, rather than an increase, of suicides [[28]].

Of course, it is no coincidence that Sweden and Trentino Alto-Adige that are heavily investing in social protections, are also characterized by a relatively egalitarian distribution of wealth and income.

## Policy implications: the need for regulations, redistribution and social protection

Policy responses following the beginning of the Great Recession in the Europe have so far, mainly consisted in two types of interventions: bank bailouts and austerity programs. On the one hand, governments adopted expensive bailouts for the “too-big-to-fail banks” exposed to toxic mortgage debts that had accumulated from reckless investments during the years of financial speculation. IMF economists estimated that, worldwide, the bailouts cost taxpayers a staggering $11.9 trillion, about a fifth of the entire globe’s annual economic output [[29]]. On the other hand, governments applied budget cuts to reduce public debts. Austerity policies revealed to be self-defeating not only because they further increased inequities in wealth, but also because they exacerbated the debt crisis they were supposed to overcome. Countries that have applied harsh budget cuts experienced sharper declines of GDP per capita and faster increases of unemployment rates [[30]]. In a recent empirical analysis, even the IMF, that championed austerity and structural adjustment programs in the developing world for over three decades, admitted that “fiscal consolidation has contractionary effects on private domestic demand and GDP” [[31]].

European governments have also failed to legislate new reforms necessary to prevent another financial downturn from occurring. John Maynard Keynes showed lucidly that although unfettered free markets have a built-in predisposition to crash at intervals, it is possible to reduce the risk of financial instability through government interventions, as demonstrated by the very low number of banking crises during the post-war era of New Deal-style policies and the Bretton Woods System. Yet, in spite of some financial reforms such as the Dodd-Frank Act in the U.S., little action has been taken to reduce systemic risk internationally. The banks that caused the 2008 financial collapse are now larger, still unregulated, full of toxic assets, and capable of neutralizing governments’ attempts to introduce more stringent financial regulations. During the New Deal, Roosevelt created a series of anti-trust laws [[32]] to reduce the power and size of “too-big-to-fail” banks. The 1936 Robinson-Patman Act, for example, delegated an anti-trust team with the task of developing policies that could, whenever necessary, break large companies into smaller entities and establish regulations to avoid the influence of large speculators on prices. In a time where banks dominate policies both in the E.U and U.S. a new set of anti-trust laws are more timely than ever.

In order to prevent future financial downturns and safeguard public health, it is also necessary to reduce wealth inequities. Recently, a group of economists have supported the Robin Hood Tax, a new Tobin Tax on speculative dealings in foreign currencies, shares and other securities of 0.05% [[33]], whose revenues can be used to support a global fund to protect vulnerable populations from the effects of the financial downturn. These policies would, in turn, reduce inequities in health. Although policy proposals continue to focus on the dichotomy budget cuts versus deficit spending, in order to promote economic stability and a healthier and more equitable Europe it is necessary to adopt some “austerity for the rich and big finance.” Recently, IMF economists have recognized that, “redistribution policies that prevent excessive household indebtedness and reduce crisis-risk ex-ante can be more desirable from a macroeconomic stabilization point of view than ex-post policies such as bailouts or debt restructurings” [[12]].

## Conclusions

The 2009 sovereign debt crisis in Europe illustrated, quite clearly, what can be the health effects experienced by societies affected by rising unemployment and financial distress. Crises can increase suicides, widen inequities in wealth and undermine the social fabric of society. The crisis is certainly not over, and prospects for economic recovery especially in countries particularly affected by the crisis such as Greece, Portugal, Italy, Ireland and Spain remain uncertain. Unless governments intervene boldly to protect the most vulnerable populations from its effects, the Eurozone crisis will continue to kill in the years to come. However, evidence showing that times of economic recessions need not to result in decreases in life expectancy, and can actually reduce mortality, is encouraging. By adopting redistributive policies and investing in strong social protection, governments can promote sustainable health and even abandon GDP as a primary national goal, to prioritize human rather than economic development. This may increasingly become necessary, if modern civilization has any serious hope to overcome the converging global ecological crises - climate change and depletion of natural resources - that threaten our future [[3]].

Of course, although policy reforms such as regulations, redistribution and social protection, and the abandonment of GDP as a primary national goal are necessary to promote a more equitable and sustainable future, it would be naïve to assume that they can be adopted under the current economic paradigm and neoliberal ideology. Measures to deal with the negative effects of the crisis on health and equity must therefore be complemented with a paradigm shift in political economy to set a new course of development where markets and profits are means to human ends, not vice versa.

## Competing interests

The author declares that he has no competing interests.
